# Oral Intake of *Hydrangea serrata* (Thunb.) Ser. Leaves Extract Improves Wrinkles, Hydration, Elasticity, Texture, and Roughness in Human Skin: A Randomized, Double-Blind, Placebo-Controlled Study

**DOI:** 10.3390/nu12061588

**Published:** 2020-05-28

**Authors:** Da-Bin Myung, Jeong-Hun Lee, Hee-Soo Han, Kwang-Young Lee, Hye Shin Ahn, Yu-Kyong Shin, Eunjung Song, Bo-Hyung Kim, Kwang Hoon Lee, Sun Hee Lee, Kyung-Tae Lee

**Affiliations:** 1 Department of Pharmaceutical Biochemistry, College of Pharmacy, Kyung Hee University, Seoul 02447, Korea; dabin_happy@naver.com (D.-B.M.); ztztzt08@hanmail.net (J.-H.L.); heesu3620@hanmail.net (H.-S.H.); gloryi@naver.com (K.-Y.L.); 2 Department of Life and Nanopharmaceutical Science, Graduate School, Kyung Hee University, Seoul 02447, Korea; 3 Department of New Material Development, COSMAXBIO, Seongnam 13486, Korea; hsahn@cosmax.com (H.S.A.); ykshin@cosmax.com (Y.-K.S.); 4 Department of Clinical Pharmacology and Therapeutics, Kyung Hee University Hospital, Seoul 02447, Korea; first0327@gmail.com (E.S.); bhkim98@khu.ac.kr (B.-H.K.); 5 East-West Medical Research Institute, Kyung Hee University, Seoul 02447, Korea; 6 Global Medical Research Center, Seoul 06035, Korea; kwanglee@yuhs.ac

**Keywords:** *Hydrangea serrata*, skin wrinkle, skin hydration, skin elasticity, skin texture, skin roughness, clinical study

## Abstract

Previously, we reported that the hot water extract of *Hydrangea serrata* leaves (WHS) and its active component, hydrangenol, possess in vitro and in vivo effects on skin wrinkles and moisturization. We conducted a randomized, double-blind, placebo-controlled trial to clinically evaluate the effect of WHS on human skin. Participants (*n* = 151) were randomly assigned to receive either WHS 300 mg, WHS 600 mg, or placebo, once daily for 12 weeks. Skin wrinkle, hydration, elasticity, texture, and roughness parameters were assessed at baseline and after 4, 8, and 12 weeks. Compared to the placebo, skin wrinkles were significantly reduced in both WHS groups after 8 and 12 weeks. In both WHS groups, five parameters (R1–R5) of skin wrinkles significantly improved and skin hydration was significantly enhanced when compared to the placebo group after 12 weeks. Compared with the placebo, three parameters of skin elasticity, including overall elasticity (R2), net elasticity (R5), and ratio of elastic recovery to total deformation (R7), improved after 12 weeks of oral WHS (600 mg) administration. Changes in skin texture and roughness were significantly reduced in both WHS groups. No WHS-related adverse reactions were reported. Hence, WHS could be used as a health supplement for skin anti-aging.

## 1. Introduction

Skin aging is divided into intrinsic aging and extrinsic aging (photoaging). The clinical characteristics of intrinsic aging are relatively minor, such as fine wrinkles, dry skin, and reduced elasticity. However, extrinsic aging causes thick and deep wrinkles when compared to intrinsic aging, and in severe cases increases skin dryness, reduces elasticity, and causes pigmental disorders and skin tumours [1]. Photoaging produces reactive oxygen species (ROS) owing to stimulation by the ultraviolet (UV) rays of sunlight and promotes the generation of pro-inflammatory cytokines that activate various signaling pathways [2]. In addition, photoaging reduces the synthesis of collagen I and collagen III, the main constituents of the dermis, by inhibiting transforming growth factor (TGF)-β and TGF-α by activating the activator protein 1 (AP-1), as well as nuclear factor kappa-light-chain-enhancer of activated B cells (NF-κB). These transcription factors promote the decomposition of intracellular tissue by activating matrix metallopeptidases (MMPs), especially MMP-1, MMP-3, and MMP-9. Aging and skin exposed to UV activates mitogen-activated protein kinases (MAPKs) to form wrinkles on the skin [3]. In skin tissues, the thickness of the epidermis is increased by two to three times during prolonged UV exposure, and in the epidermal layer, this is characterized by an increase in the number of prickle cells, the polymorphism of keratinocytes. Moreover, substances, including an amorphous elastosis, are produced in the dermis and dermal connective layers, where collagen and elastosis are deformed, increasing the elastic fibers. The elasticity or moisturization of the skin is maintained by the collagen present in the skin’s outermost stratum corneum and the dermis. The moisture content of the stratum corneum is determined by the lipid mixture produced by the epidermis and by the natural moisturizing factor (NMF), a water-soluble component present in each layer [4]. The keratin layer of the healthy epidermis contains 15–20% water, and if the water drops below 10%, the skin will get dehydrated, and the wrinkles will increase as it loses its gloss and elasticity.

Hydrangea plants have a variety of bioactive compounds such as dihydroisocoumarins, secoiridoids, and stilbenes (hydrangenol, phyllodulcin, macrophylloside, and their glucosides) [5,6]. Among these compounds, we previously reported that hydrangenol, a dihydroisocoumarin, possesses potential protective effects on cell viability, production of procollagen type I, MMP-1, and pro-inflammatory cytokines [7]. Moreover, it has been reported that hydrangenol exhibits anti-inflammatory [8], anti-microbial [9], anti-diabetic [10], anti-allergic [11], antimalarial [12], and anti-cancer activities [13,14,15]. It has been reported that the scheme for the biosynthesis of hydrangenol with the aid of C^14^-labelled compounds was found to be the condensation of an intact phenylpropanoid molecule with three molecules of acetate [16,17]. In our previous study, a hot water extract of *Hydrangea serrata* leaves (WHS) has shown to protect ultraviolet B (UVB)-induced cell viability and production of pro-collagen type I and hyaluronic acid (HA) following in vitro and oral administration of WHS reduced the area, length, and depth of wrinkles in a UVB-irradiated mouse model. Furthermore, the increased skin thickness observed in the UVB group was reduced following the administration of WHS, and collagen fiber density and pro-collagen type I production recovered [18]. Based on these results, the clinical evaluation was designed to verify the efficacy and safety of WHS (daily oral dosage of 300 or 600 mg of WHS for 12 weeks in 151 volunteers) on skin wrinkle improvement and moisturization.

## 2. Materials and Methods

### 2.1. Preparation of Test Material and Determination of Dose

The dried leaves of *H. serrata* were extracted with distilled water at 98 °C for 5 h followed by filtration and then spray-dried to give a dried extract residue WHS with a yield of 23% (*w*/*w*), which contained 7.7 mg/g of hydrangenol as the bioactive compound. One test tablet contained 300 mg or 600 mg WHS composed of agents (Table S1). The placebo tablet which was indistinguishable from the test tablet.

In our previous animal study using hairless (HR)-1 mice, WHS exerted its effects on skin wrinkle, skin hydration, transepidermal water loss (TEWL), collagen formation in the dermis, and the regulation of MMPs expression at the dose of 50 mg/kg/day and 100 mg/kg/day [18]. Based on the doses of efficacy in the UVB-irradiated hairless mice, this is converted to human equivalent dose (HED) results in 4.05 mg/kg and 8.1 mg/kg, in other words 243 mg/day and 486 mg/day for a person weighing 60 kg [19]. In addition, single toxicity test confirmed that hot water extract of *Hydrangea serrata* leaves (WHS) did not show any toxicity (>5000 mg/kg). Based on a comprehensive review of the single toxicity test intake and the amount of pharmacologically active dose (PAD) intake, the daily intake of this human application test was set at 300 mg/day and 600 mg/day, as it was deemed safe to proceed up to 600 mg/day for the human application test.

### 2.2. Study Design

This clinical study had a randomized, double-blind, placebo-controlled design. It was conducted according to the applicable Global Medical Research Center (17th floor, 107 Dosan-daero, Gangnam-gu, Seoul, Korea) from 30 November 2018, to 31 May 2019. The study protocol was reviewed and approved by the institutional review board of COSMAX BIO (Protocol No.: COSMAX BIO_WHS). This human application study was conducted in accordance with the International Clinical Trial Management Standard (ICH GCP).

### 2.3. Study Participants

Healthy male and female aged 35–60 years (*n* = 151) who volunteered and met specified inclusion and exclusion criteria were recruited for the study (Table S2). Inclusion criteria included global skin wrinkle grade higher than score 3, as determined by dermatologists according to the “Guideline for Efficacy Evaluation of Functional Cosmetics” published by Korean Ministry of Food and Drug Safety (KMFDS) [20]. Before proceeding to the clinical study, the participants were informed clearly and precisely of the objective and the protocol of the study, and of foreseeable risks involved in the trial. All participants voluntarily signed written informed consent form. Two individuals withdrew consent, and the remaining 149 commenced the study. Three participants dropped out of the study for personal reasons, and 146 completed the study (Figure S1).

### 2.4. Study Schedule

**Figure 1:**
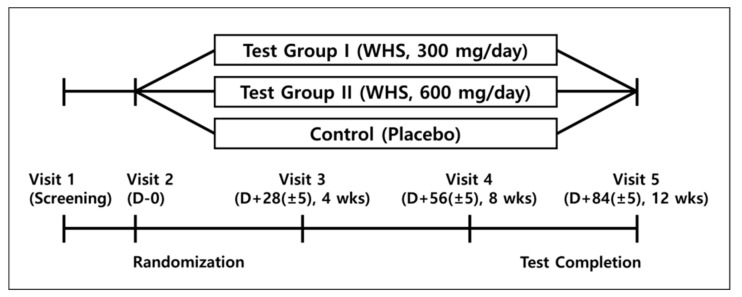
Clinical study overview.

After determination whether they meet the inclusion or exclusion criteria through screening visit, and then randomly assigned to either WHS 300 mg group (test group I), WHS 600 mg group (test group II), or placebo group (control group). Assigned subjects consumed test I, II or control food for 12 weeks (Figure 1).

### 2.5. Measurement of Skin Wrinkle

Skin wrinkle was assessed by two methods: visual assessment measured by Mark-Vu (PSIPLUS Co. LTD, Suwon, Korea) by dermatologists and instrumental analysis of skin-replica images. For visual assessment, cutaneous examinations of the crow’s-feet area were conducted by dermatologist with a double-blind method based on a “Guideline for Efficacy Evaluation of Functional Cosmetics” published by KMFDS (Cheongju, Korea) [20]. For instrumental analysis of skin-replica images, replicas made with a silicone-based solution and a catalyst (Courage and Khazaka, Köln, Germany) were taken from the designated crow’s-feet area and analyzed with a Skin Visiometer SV 700 (Courage and Khazaka) that evaluates the topography of the skin surface by light transmission of a very thin silicone replica based on skin-wrinkle parameters: R1 (skin roughness), R2 (maximum roughness), R3 (average roughness), R4 (smoothness depth), and R5 (arithmetic average roughness).

### 2.6. Measurement of Skin Hydration and TEWL

Hydration and TEWL of the skin of the crow′s-feet area of the designated side of the face were measured with a Corneometer CM 825 (Courage and Khazaka, Köln, Germany) and Tewamater TM 300 (Courage and Khazaka, Köln, Germany), respectively.

### 2.7. Measurement of Skin Elasticity

A Cutometer MPA 580 (Courage and Khazaka, Köln, Germany) was used for assessment of skin elasticity of the designated crow′s-feet area, based on suction of the skin using a probe with negative pressure of 450 mbar, which makes the test area drawn into the aperture of the probe. Measurement was repeated three times with 2 s of suction time followed by 2 s of relaxation time for each measurement. Curves of skin deformation obtained were analyzed with Win Cutometer MPA software to obtain the values of skin-elasticity parameters: R2 (overall elasticity of the skin), R5 (net elasticity), and R7 (the ratio of elastic recovery to total deformation).

### 2.8. Measurement of Skin Texture and Roughness

Skin texture of the skin of cheek area and skin roughness of the skin of crow’s-feet area of the designated side of the face were measured with an Antera 3D camera for skin analysis (CS) (Miravex, Dublin, Ireland).

### 2.9. Safety Assessment

Safety tests conducted at visit 1 (0 weeks) and visit 5 (12 weeks) were hematological tests, blood-chemical tests, urine tests, and vital-sign measurements. Hematological tests parameters were white blood cell (WBC), red blood cell (RBC), hemoglobin (Hb), hematocrit (Hct), platelet, neutrophil, lymphocyte, monocyte, eosinophil, and basophil. Blood-chemical test items were glucose, total protein, albumin, total bilirubin, blood urea nitrogen (BUN), aspartate aminotransferase (AST), alanine aminotransferase (ALT), γ-guanosine triphosphate (γ-GTP), creatinine, total cholesterol, high density lipoprotein (HDL)-cholesterol, low density lipoprotein (LDL)-cholesterol, triglyceride, and thyroid stimulating hormone (TSH). Urine test items were specific gravity (SG), pH, protein, glucose, ketone, bilirubin, blood, urobilinogen, nitrite, and WBC. Vital signs and somatometry measured were pulse, systolic and diastolic blood pressures, and weight.

### 2.10. Statistical Analysis

Statistical analyses were performed using SAS^®^ (Version 9.4, SAS Institute, Cary, NC, USA). When the normality was satisfied, the paired *t*-test and two-sample *t*-test were used to determine the significance of differences within groups and between groups, respectively. However, data were analyzed using the Wilcoxon signed-rank test and Wilcoxon rank-sum test for comparisons within groups and between two groups, respectively when normality assumption was violated. Data are presented as mean ± standard deviation (SD). Each statistical testing was conducted at significance level 0.05.

## 3. Results

### 3.1. Baseline Characteristics (Vital Signs and Somatometry) of Participants

Participants (*n* = 151) were randomized at baseline and allocated to the WHS 300 mg group (*n* = 50), WHS 600 mg group (*n* = 50), or the placebo group (*n* = 51). At baseline, age, weight, systolic and diastolic blood pressures, and pulse demonstrated no significant difference between the WHS groups and the placebo group (Table S3).

### 3.2. Effect of WHS on Skin Wrinkle

**Figure 2:**
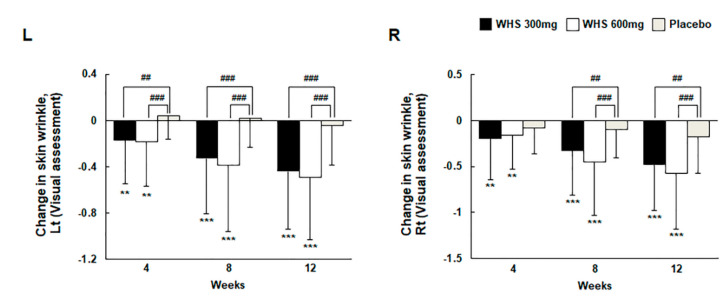
Changes in skin wrinkle (visual evaluation) in the *Hydrangea serrata* leaves (WHS) 300 mg group, WHS 600 mg group, or placebo group after 12 weeks. * indicates compared within groups’; *p*-value (** *p* < 0.01 and *** *p* < 0.001) for Wilcoxon signed-rank test. ^#^ indicates compared between WHS 300 mg group and placebo group, WHS 600 mg group and placebo group; *p*-value (^##^ *p* < 0.01 and ^###^ *p* < 0.001) using the Wilcoxon rank-sum test.

**Figure 3:**
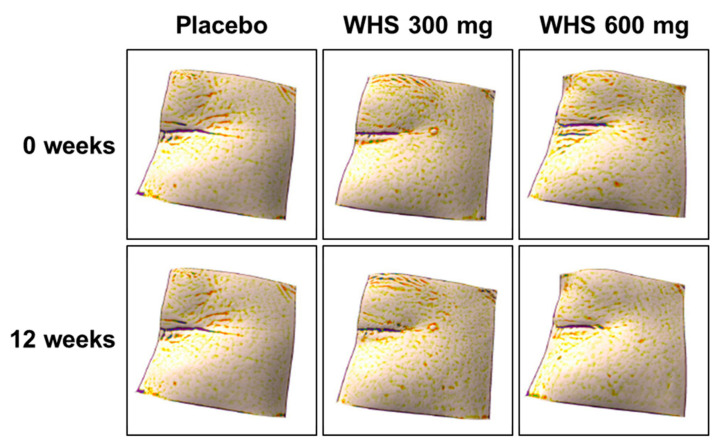
Representative crow’s feet 3D images in the WHS 300 mg group, WHS 600 mg group, or placebo group after 12 weeks.

The analysis of skin wrinkle changes in both left (Lt) and right (Rt) crow’s feet, measured by visual assessment and Mark-Vu after 12 weeks of oral intake, present the grade of change from the baseline value in skin wrinkle and are summarized as the mean ± SD. The summarized statistics values were −0.44 ± 0.50 (Lt) and −0.48 ± 0.50 (Rt) in the WHS 300 mg group, −0.49± 0.54 (Lt) and −0.57 ± 0.61 (Rt) in the WHS 600 mg group, and −0.04 ± 0.35 (Lt) and −0.18 ± 0.39 (Rt) in the placebo group. For the WHS 300 mg and the WHS 600 mg groups, the grade of changes from the baseline was significantly lower than those observed for the placebo group at 12 weeks (Figure 2 and Figure 3).

Moreover, skin-wrinkling parameters were measured using the Skin Visiometer SV700, 2 cm from the tail of the left or right eye. Five parameters were measured in both test areas: R1 (Skin roughness), R2 (Maximum roughness), R3 (Average roughness), R4 (Smoothness depth), and R5 (Arithmetic average roughness). The results of the analysis for skin-wrinkling parameters are summarized in Table 1. After 12 weeks of oral intake, the changes in parameters from the baseline value for R1 were −0.06 ± 0.06 (Lt) and −0.07 ± 0.05 (Rt) in the WHS 300 mg group and −0.06 ± 0.05 (Lt) and −0.05 ± 0.05 (Rt) in the WHS 600 mg group. However, the R1 value in the placebo group increased by 0.01 ± 0.06 (Lt) and 0.01 ± 0.04 (Rt). In the case of R2 values, the changes from the baseline value were −0.05 ± 0.02 (Lt) and −0.05 ± 0.03 (Rt) in the WHS 300 mg group and −0.05 ± 0.03 (both) in the WHS 600 mg group, after 12 weeks of oral intake. However, the R2 value increased by 0.01 ± 0.02 (both) in the placebo group. In R3 values, the changes from baseline values were −0.03 ± 0.01 (both) in the WHS 300 mg group and −0.03 ± 0.02 (both) in the WHS 600 mg group, after 12 weeks of oral intake. However, the R3 value in the placebo group increased by 0.01 ± 0.01 (both). In the case of R4 values, the changes from baseline value were −0.02 ± 0.05 (Lt) and −0.03 ± 0.04 (Rt) in the WHS 300 mg group and −0.03 ± 0.03 (Lt) and −0.03 ± 0.09 (Rt) in the WHS 600 mg group, after 12 weeks of oral intake. However, the R4 value in the placebo group increased by 0.01 ± 0.05 (Lt) and 0.01 ± 0.03 (Rt). In the case of the R5 values, the changes from baseline values were −0.01 ± 0.02 (Lt) and −0.01 ± 0.01 (Rt) in the WHS 300 mg group and −0.01 ± 0.01 (both) in the WHS 600 mg group, after 12 weeks of oral intake; however, the R5 value in the placebo group increased by 0.00 ± 0.02(Lt) and 0.00 ± 0.01(Rt). For both WHS dose groups, the changes for each parametric value (R1-R5) from the baseline were significantly lower than those observed for each value of the placebo group, except for the change in the R5 value measured in the tail of the left eye of the WHS 300 mg group.

### 3.3. Effect of WHS on Skin Hydration

**Figure 4:**
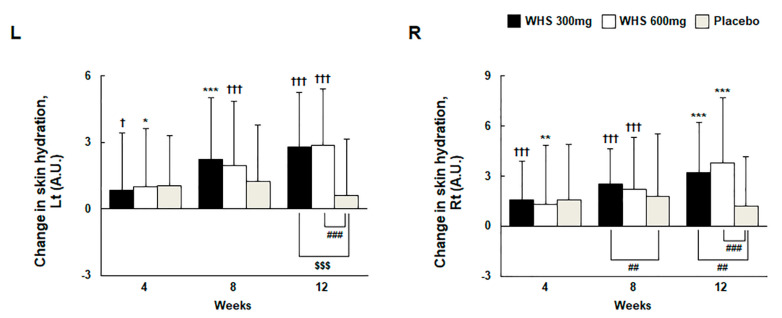
Changes in skin hydration with WHS or placebo administration for 12 consecutive weeks. Skin hydration was measured with a Corneometer CM825. ^†^ indicates compared within groups; *p*-value (^†^ *p* < 0.05 and ^†††^ *p* < 0.001) for paired *t*-test. * indicates compared within groups; *p*-value (* *p* < 0.05, ** *p* < 0.01, and *** *p* < 0.001) for Wilcoxon signed-rank test. ^$^ indicates compared between WHS 300 mg group and placebo group, WHS 600 mg group and placebo group; *p*-value (^$$$^ *p* < 0.001) by two-sample *t*-test. ^#^ indicates compared between WHS 300 mg group and placebo group, WHS 600 mg group and placebo group; *p*-value (^##^ *p* < 0.01 and ^###^ *p* < 0.001) using the Wilcoxon rank-sum test.

Skin hydration was measured using the Corneometer CM825 at the perpendicular intersection between the tip of the left or right eye and nose. For skin moisture content, the change from baseline after 12 weeks of oral intake was 2.78 ± 2.48 AU (Lt) and 3.20 ± 3.01 AU (Rt) in the WHS 300 mg group, 2.87 ± 2.53 AU (Lt) and 3.81 ± 3.88 AU (Rt) in the WHS 600 mg group, and 0.61 ± 2.52 AU (Lt) and 1.21 ± 2.95 AU (Rt) in the placebo group (Figure 4). On comparing with the placebo group, skin hydration was significantly enhanced in the WHS 300 mg group, as well as the WHS 600 group (both, *p* < 0.001), following the 12-week treatment. The measurement of TEWL using the Tewameter TM300 was performed at the perpendicular intersection between the tip of the left or right eye and nose. However, no significant changes were observed in the TEWL after 12 weeks of oral WHS intake when compared with the placebo group (Figure S2).

### 3.4. Effect of WHS on Skin Elasticity

The measurement of skin elasticity using the Cutometer MPA580 was performed at the perpendicular intersection between the left or right eye pupil and nose tip (Table 2). Three parameters were measured: R2 (Ua/Uf; the overall elasticity of the skin, including creep and creep recovery), R5 (Ur/Ue; the net elasticity), and R7 (Ur/Uf; the ratio of elastic recovery to the total deformation). In each parameter, the closer the value is to 1 (100%), the greater the skin elasticity. After 12 weeks of oral WHS intake, the R2 value was altered from baseline to 0.018 ± 0.028 (Lt) and 0.008 ± 0.027 (Rt) in the WHS 300 mg group, 0.017 ± 0.030 (Lt) and 0.019 ± 0.032 (Rt) in the WHS 600 mg group, and 0.005 ± 0.026 (Lt) and 0.004 ± 0.024 (Rt) in the placebo group. In both WHS groups, the changes in the R2 values at 12 weeks were significantly greater than those observed for the placebo group, except for the changed R2 value measured in the right area of the WHS 300 mg group. In the WHS 600 mg, the changes in the R5 values in the left area at eight weeks and right area at 12 weeks were significantly greater than those in the placebo group (*p* < 0.05). However, changes in the R5 value in both test areas of the WHS 300 mg group were not significantly greater than those observed in the placebo group. In the case of the placebo group, the change in the R5 value, from baseline to 12 weeks, was −0.037 ± 0.078 (Lt) and −0.054 ± 0.072 (Rt). In the WHS 600 mg group, the change in the R7 value measured in the left test area was not significant when compared with that observed in the placebo group after 12 weeks. However, the change in R7 value measured in the right test area of the WHS 600 mg group was significantly greater than that in the placebo group (*p* < 0.05) after 12 weeks. In the placebo group, the altered R7 value was −0.007 ± 0.039 (Lt) and −0.014 ± 0.035 (Rt).

### 3.5. Effect of WHS on Skin Texture and Roughness

**Figure 5:**
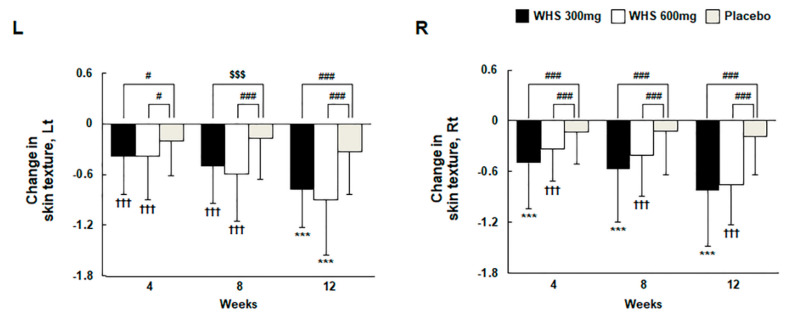
Changes in skin texture with WHS or placebo administration for 12 consecutive weeks. Skin texture was measured with an Antera 3D camera for skin analysis (CS). ^†^ indicates compared within groups; *p*-value (^†††^ *p* < 0.001) for paired *t*-test. * indicates compared within groups; *p*-value (^***^ *p* < 0.001) for Wilcoxon signed-rank test. ^$^ indicates compared between WHS 300 mg group and placebo group, WHS 600 mg group and placebo group; *p*-value (^$$$^ *p* < 0.001) by two-sample *t*-test. ^#^ indicates compared between WHS 300 mg group and placebo group, WHS 600 mg group and placebo group; *p*-value (^#^ *p* < 0.05 and ^###^ *p* < 0.001) using the Wilcoxon rank-sum test.

**Figure 6:**
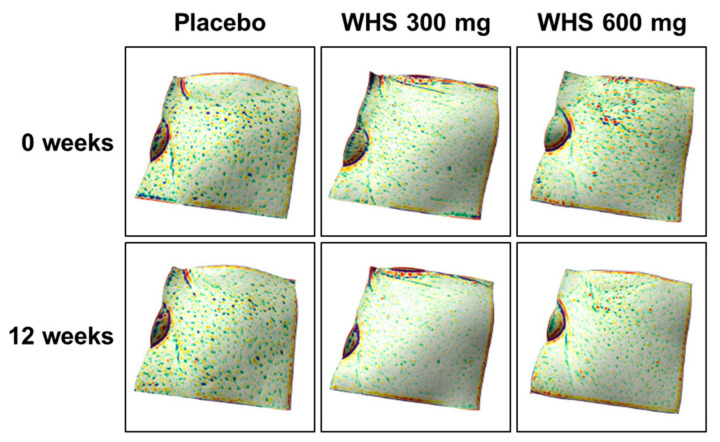
Representative skin texture 3D images with WHS or placebo group after treatment for 12 consecutive weeks.

**Figure 7:**
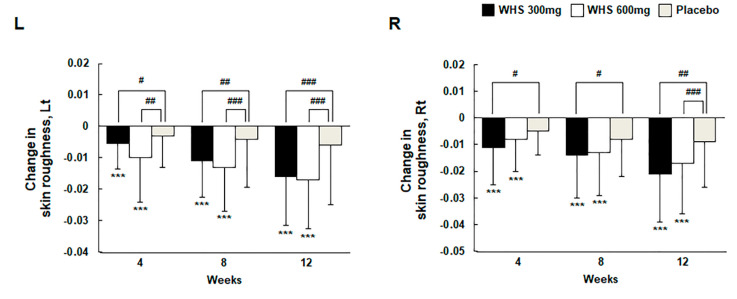
Changes in skin roughness with WHS or placebo after administration for 12 consecutive weeks. Skin roughness was measured with an Antera 3D CS. * indicates compared within groups; *p*-value (*** *p* < 0.001) for Wilcoxon signed-rank test. ^#^ indicates compared between WHS 300 mg group and placebo group, WHS 600 mg group and placebo group; *p*-value (^#^ *p* < 0.05, ^##^ *p* < 0.01, and ^###^ *p* < 0.001) using the Wilcoxon rank-sum test.

Skin texture was measured using the Antera 3D CS in the left or right cheek area beside the nose. After 12 weeks of oral intake, the changes of skin texture were −0.77 ± 0.46 (Lt) and −0.82 ± 0.66 (Rt) in the WHS 300 mg group, −0.90 ± 0.65 (Lt) and −0.76 ± 0.47 (Rt) in the WHS 600 mg group, and −0.33 ± 0.51 (Lt) and −0.19 ± 0.45 (Rt) in the placebo group (Figure 5 and Figure 6). For both WHS dose groups, the changes in skin texture from the baseline were significantly lower than those in the placebo group (both, *p* < 0.001). The measurement of skin roughness by Antera 3D CS was performed 2 cm away from the tail of the left or right eye. After 12 weeks of oral intake, the change in skin roughness were −0.016 ± 0.016 (Lt) and −0.021 ± 0.018 (Rt) in the WHS 300 mg group, −0.017 ± 0.016 (Lt) and −0.017 ± 0.019 (Rt) in the WHS 600 mg group, and −0.006 ± 0.019 (Lt) and −0.009 ± 0.017 (Rt) in the placebo group (Figure 7). For both WHS dose groups, the changes in skin roughness from the baseline after 12 weeks were significantly (*p* < 0.001) lower than those observed in the placebo group, except for the changed R5 values measured in the right area of the WHS 300 mg group (*p* < 0.01).

### 3.6. Analysis of Laboratory Parameters and Adverse Reactions

No severe adverse effects were reported during the entire study period. Furthermore, no abnormal, serious, or delayed systemic symptoms or signs were observed in relation to the test product during its application period.

## 4. Discussion

Nutricosmetics and health functional food define edible natural health products, including natural extracts that support to improve the function and appearance of human skin, hair, nails, and reduce body fat. Additionally, the aforementioned are agents that enhance factors governing external appearance by providing the body’s internal system with protective or reactive mechanisms, and then offer beautification and/or personal hygiene benefits. In 2025, the health functional food market will reach 275.8 billion, with a combined average growth rate of 7.9% during the forecasted period [21]. This positive trend is due to the consumers’ awareness of aesthetic demands, skin aging, and wrinkle formation in an aging society.

Generally, the major target of nutricosmetics is the anti-aging, skin, and body care product market. Plant extracts have been used for cosmetics and skincare products since ancient times, as a complex mixture of natural compounds with different structures and functions. Reportedly, various plant extracts, such as fermented barley and soybean mixtures [22], horsetail (*Equisetum arvense* L.) [23], *Aloe vera* [24,25], pine bark (*Pinus pinaster*), *Citrus paradisi* and *Rosmarinus officinalis* extracts, red-orange complex [26], and *Polypodium leucotomos* extract [27] are effective skincare agents in animal or clinical studies. Additionally, the fermented honeybush (*Cyclopia intermedia*) extract (HU-018) 400 mg or 800 mg, currently released as a health functional food, reduces skin wrinkles, improves skin elasticity, and enhances skin moisture [28]. 

Hydrangea leaves have been consumed as traditional herbal tea and medicine in its native far-east Asian countries, including Korea, China, and Japan, and are known to have a distinctive mint-flavored sweet taste [29]. Hydrangea is native to the Korean mountains known as “San-su-guk, Mountain Hydrangea, or Tea of Heaven.” Our phytochemical study showed that a variety of compounds (hydrangenol, thunberginol A, thunberginol C, hydrangenoside A, hydrangenoside C, cudrabibenzyl A, 2,3,4′-trihydroxystilbene, thunberginol F, quercetin 3-*O*-*β*-D-xylopyranosyl (1–2)-*β*-D-galactopyranoside, quercetin 3-*O*-*β*-Dxylopyranosyl (1–2)-*β*-D-glucopyranoside, cudrabibenzyl A, 2,3,4′-trihydroxystilbene, quercetin 3-*O*-*β*-Dxylopyranosyl (1–2)-*β*-D-galactopyranoside, and quercetin 3-*O*-*β*-D-xylopyranosyl (1–2)-*β*-D-glucopyranoside) isolated from *H. serrata* possess anti-photoaging activity in UVB-exposed human fibroblasts [7]. Of these various compounds, hydrangenol was found to possesses potential protective effects on anti-photoaging properties in vitro and in vivo and then used as an active indicator compound [7,30]. Based on its anti-photoaging and anti-inflammatory activities, it is expected that hydrangenol could be used as an anti-photoaging agent. In Hs68 human foreskin fibroblasts and hairless mice, WHS, which contains hydrangenol as a main component, suppresses the expression of photoaging markers induced by UVB irradiation. Moreover, WHS regulates the expression of MMPs and collagen by inhibiting UVB-induced activation of MAPK, AP-1, and signal transducer and activator of transcription (STAT1) signaling pathways in Hs68 and hairless mice. In the UVB-induced photoaging mouse model, oral WHS administration reportedly alleviates the total area, mean length, mean depth, and maximal depth of skin wrinkles [18]. Hence, we evaluated the anti-aging effect of oral WHS intake in a randomized, double-blind, and placebo-controlled trial. In this clinical trial, the results of in vitro and in vivo experiments conducted have been verified. We aimed to confirm the optimal dose for the oral administration of WHS and based on the results of adverse reactions, WHS was safe in subjects at the dosage used in this study. In terms of the single-dose toxicity, the result of acute toxicity demonstrates that oral WHS did not induce mortality or toxicity up to 5000 mg/kg in rats. In this clinical trial, we investigated the efficacy of oral WHS intake in human skin functions. The results demonstrated that the oral ingestion of WHS 300 mg and 600 mg once daily for 12 weeks significantly improved skin wrinkles, hydration, elasticity, texture, and roughness in the participants.

In this study, the improvement in skin wrinkles following WHS intake is consistent with the previous study, in which the expression of MMPs (MMP-1 and MMP-3) is downregulated by oral WHS administration in UVB-irradiated mice, thereby increasing the collagen content in the skin and reducing wrinkle formation [18]. Furthermore, WHS increases HA production and epidermal water content in the animal study and reduces TEWL [18]. Moreover, a similar trend has been observed with hydrangenol, an effective indicator component of WHS [30]. Hydrangenol downregulates hyaluronidases (HYALs), which perform to decompose HA in the Hs68 human foreskin fibroblasts and UVB-irradiated hairless mice [7,30]. In addition, aquaporin-3, which serves to transport water and glycerol in the skin [31], is upregulated by hydrangenol in an animal model [30]. Therefore, it may be hypothesized that, in this study, the improvement in skin hydration mediated by WHS could be attributed to hydrangenol present in WHS. Unlike the results of the previous animal study, WHS demonstrated no effect on TEWL in this clinical study. In the case of skin elasticity, WHS improved the parameters R2, R5, and R7, and these results may be consistent with our previous results, inhibiting UVB-induced elastin degradation by reducing elastase activity.

Recently, interest in the development of anti-aging agents for the skin, photoprotective oral dietary supplements based on plant extracts and natural compounds, and cosmeceuticals has been increasing [32]. In particular, WHS or its ingredient is the dominant activator that possesses protective functions against dermal dehydration or wrinkle formation. Their anti-photoaging effects have been demonstrated in vitro and in vivo, but species differences may exist. Therefore, it appeared that the efficacy of pre-clinical and clinical trials could differ. Although the underlying mechanisms by which WHS improves skin wrinkles, hydration, elasticity, texture, and roughness were not investigated in this clinical trial, we suggest that the WHS supplement may have a positive effect on collagen decomposition based on the previous in vitro and in vivo studies [7]. Additionally, these preventive skin aging effects of WHS may be mediated through the anti-oxidative activities of hydrangenol, an active-constituent of *H. serrata*. As WHS may not reach the dermal layer where collagen, elastin, and extracellular matrix exist, isolated topical administration may not be efficacious in restoring damaged or aged skin. Hence, nutricosmetic products should be consumed, given that the extracts and their active compounds can be absorbed, and several signal transductions can be regulated within the cells of the target tissue.

In conclusion, our randomized, double-blinded, placebo-controlled study demonstrated that oral WHS supplements produced significant anti-aging effects. Therefore, WHS has potential as a dietary supplement to protect against skin aging in the health functional food, targeting systemic factors regulating skin appearance.

## Figures and Tables

**Table 1:** Changes of skin-wrinkle parameters measured by Skin Visiometer SV700.

**Left**	**Evaluation Parameter**	**Time Point**	**WHS 300 mg Group**				**WHS 600 mg Group**				**Placebo Group**	
			**Mean**	**(SD)**	**Test/Placebo** ***p*-Value**		**Mean**	**(SD)**	**Test/Placebo** ***p*-Value**		**Mean**	**(SD)**
	**R1**	4 W	−0.01	(0.06)	0.133		−0.03	(0.05)†††	<0.001	###	0.01	(0.05)
		8 W	−0.04	(0.06) †††	<0.01	$$	−0.05	(0.06) †††	<0.001	###	0.00	(0.06)
		12 W	−0.06	(0.06) †††	<0.001	###	−0.06	(0.05) †††	<0.001	###	0.01	(0.06)
	**R2**	4 W	−0.01	(0.03) †	<0.050	$	−0.02	(0.02) †††	<0.001	###	0.00	(0.02)
		8 W	−0.03	(0.02) †††	<0.001	###	−0.03	(0.03) ***	<0.001	###	0.01	(0.03)
		12 W	−0.05	(0.02) †††	<0.001	$$$	−0.05	(0.03) ***	<0.001	###	0.01	(0.02)
	**R3**	4 W	−0.01	(0.01) ***	<0.001	###	−0.01	(0.01) ***	<0.001	###	0.00	(0.01)
		8 W	−0.02	(0.01) ***	<0.001	###	−0.02	(0.02) ***	<0.001	###	0.00	(0.01)
		12 W	−0.03	(0.01) ***	<0.001	###	−0.03	(0.02) ***	<0.001	###	0.01	(0.01)
	**R4**	4 W	0.00	(0.05)	0.595		−0.02	(0.04) †	<0.05	#	0.00	(0.04)
		8 W	−0.01	(0.05) †	0.173		−0.02	(0.04) †††	<0.05	#	0.00	(0.05)
		12 W	−0.02	(0.05) ††	<0.01	##	−0.03	(0.03) †††	<0.001	###	0.01	(0.05)
	**R5**	4 W	0.00	(0.02)	0.595		−0.01	(0.02) **	0.141		0.00	(0.02)
		8 W	0.00	(0.02)	0.252		−0.01	(0.01) ***	< 0.01	##	0.00	(0.02)
		12 W	−0.01	(0.02) †	0.063		−0.01	(0.01) ***	< 0.01	##	0.00	(0.02)
**Right**	**Evaluation Parameter**	**Time Point**	**WHS 300 mg Group**				**WHS 600 mg Group**				**Placebo Group**	
			**Mean**	**(SD)**	**Test/Placebo** ***p*-Value**		**Mean**	**(SD)**	**Test/Placebo** ***p*-Value**		**Mean**	**(SD)**
	**R1**	4 W	−0.04	(0.05) ***	<0.001	###	−0.02	(0.04) ††	<0.01	##	0.01	(0.05)
		8 W	−0.05	(0.05) †††	<0.001	$$$	−0.04	(0.05) ***	<0.001	###	0.01	(0.05)
		12 W	−0.07	(0.05) †††	<0.001	$$$	−0.05	(0.05) †††	<0.001	###	0.01	(0.04)
	**R2**	4 W	−0.02	(0.03) ***	<0.010	$$	−0.02	(0.03) ***	<0.01	##	0.00	(0.02)
		8 W	−0.03	(0.03) ***	<0.001	###	−0.03	(0.03) ***	<0.001	###	0.00	(0.02)
		12 W	−0.05	(0.03) ***	<0.001	###	−0.05	(0.03) ***	<0.001	###	0.01	(0.02)
	**R3**	4 W	−0.01	(0.01) ***	<0.001	###	−0.01	(0.02) ***	<0.001	###	0.00	(0.01)
		8 W	−0.02	†††	<0.001	###	−0.02	(0.01) ***	<0.001	###	0.00	(0.01)
		12 W	−0.03	(0.01) ***	<0.001	###	−0.03	(0.02) ***	<0.001	###	0.01	(0.01)
	**R4**	4 W	−0.02	(0.04) †††	<0.001	###	−0.02	(0.09) *	<0.01	##	0.01	(0.04)
		8 W	−0.03	(0.04) ***	<0.001	###	−0.03	(0.09) *	<0.05	#	0.00	(0.04)
		12 W	−0.03	(0.04) †††	<0.001	$$$	−0.03	(0.09) ***	<0.001	###	0.01	(0.03)
	**R5**	4 W	−0.01	(0.01) ***	<0.001	###	0.00	(0.01)	0.128		0.00	(0.01)
		8 W	−0.01	(0.01) ***	<0.001	###	0.00	(0.01) *	0.068		0.00	(0.01)
		12 W	−0.01	(0.01) ***	<0.001	###	−0.01	(0.01) **	<0.01	##	0.00	(0.01)

Skin wrinkling parameters: R1, Skin roughness; R2, Maximum roughness; R3, Average roughness; R4, Smoothness depth; R5, Arithmetic average roughness. ^†^ Compared within groups; *p*-value (^†^ *p* < 0.05, ^††^ *p* < 0.01, and ^†††^ *p* < 0.001) for paired *t*-test. * Compared within groups; *p*-value (* *p* < 0.05, ** *p* < 0.01, and *** *p* < 0.001) for Wilcoxon signed-rank test. ^$^ Compared between WHS 300 mg group and placebo group, WHS 600 mg group and placebo group; *p*-value (^$^ *p* < 0.05, ^$$^ *p* < 0.01, and ^$$$^ *p* < 0.001) by two-sample *t*-test. ^#^ Compared between WHS 300 mg group and placebo group, WHS 600 mg group and placebo group; *p*-value (^#^ *p* < 0.05, ^##^ *p* < 0.01, and ^###^ *p* < 0.001) by Wilcoxon rank-sum test.

**Table 2:** Changes of skin elasticity measured by Cutometer MPA580.

**Left**	**Evaluation Parameter**	**Time Point**	**WHS 300 mg Group**				**WHS 600 mg Group**				**Placebo Group**	
			**Mean**	**(SD)**	**Test/Placebo** ***p*-Value**		**Mean**	**(SD)**	**Test/Placebo** ***p*-Value**		**Mean**	**(SD)**
	**R2** **(Ua/Uf)**	4 W	0.009	(0.024) †	0.173		0.008	(0.028) †	0.248		0.002	(0.024)
		8 W	0.013	(0.027) **	0.05		0.012	(0.028) ††	0.052		0.001	(0.023)
		12 W	0.018	(0.028) †††	<0.05	$	0.017	(0.030) †††	<0.05	#	0.005	(0.026)
	**R5** **(Ur/Ue)**	4 W	0.007	(0.079)	0.358		0.002	(0.072)	0.520		−0.007	(0.068)
		8 W	0.010	(0.093)	0.105		0.013	(0.076)	<0.05	#	−0.017	(0.063)
		12 W	−0.009	(0.086)	0.215		−0.007	(0.076)	0.076		−0.037	(0.078)
	**R7** **(Ur/Uf)**	4 W	0.009	(0.046)	0.473		0.010	(0.039)	0.309		0.002	(0.036)
		8 W	0.012	(0.054)	0.156		0.012	(0.040) †	0.062		−0.001	(0.032)
		12 W	0.009	(0.048)	0.072		0.007	(0.045)	0.121		−0.007	(0.039)
**Right**	**Evaluation Parameter**	**Time Point**	**WHS 300 mg group**				**WHS 600 mg Group**				**Placebo Group**	
			**Mean**	**(SD)**	**Test/Placebo** ***p*-Value**		**Mean**	**(SD)**	**Test/Placebo** ***p*-Value**		**Mean**	**(SD)**
	**R2** **(Ua/Uf)**	4 W	0.002	(0.021)	0.057		−0.001	(0.028)	0.175		−0.007	(0.035)
		8 W	0.011	(0.026) ††	<0.05	#	0.007	(0.028) *	0.202		0.004	(0.025)
		12 W	0.008	(0.027) †	0.419		0.019	(0.032) †††	<0.05	#	0.004	(0.024)
	**R5** **(Ur/Ue)**	4 W	−0.019	(0.066)	0.354		−0.010	(0.072)	0.144		−0.033	(0.083)
		8 W	−0.011	(0.080)	0.218		−0.009	(0.088)	0.177		−0.030	(0.066)
		12 W	−0.042	(0.076) ***	0.325		−0.017	(0.073)	<0.05	#	−0.054	(0.072) ***
	**R7** **(Ur/Uf)**	4 W	−0.009	(0.032) †	0.635		−0.001	(0.041)	0.164		−0.013	(0.049)
		8 W	−0.004	(0.041)	0.281		0.001	(0.047)	0.260		−0.009	(0.034)
		12 W	−0.015	(0.043) †	0.962		0.004	(0.044)	<0.05	#	−0.014	(0.035)

Skin-elasticity parameters: R2, overall elasticity; R5, net elasticity; R7, ratio of elastic recovery to total deformation. ^†^ indicates compared within groups; *p*-value (^†^ *p* < 0.05, ^††^ *p* < 0.01, and ^†††^ *p* < 0.001) for paired *t*-test. * indicates compared within groups; *p*-value (* *p* < 0.05, ** *p* < 0.01, and *** *p* < 0.001) for Wilcoxon signed-rank test. ^$^ indicates compared between WHS 300 mg group and placebo group, WHS 600 mg group and placebo group; *p*-value (^$^ *p* < 0.05) by two-sample *t*-test. ^#^ indicates compared between WHS 300 mg group and placebo group, WHS 600 mg group and placebo group; *p*-value (^#^ *p* < 0.05) using the Wilcoxon rank-sum test.

## Supplementary Materials

The following are available online at https://www.mdpi.com/2072-6643/12/6/1588/s1, Figure S1: The study flow diagram, Figure S2: Changes in transepidermal water loss (TEWL) in taking WHS or placebo following 12 consecutive weeks. TEWL was measured with a Tewameter TM300, Table S1: Ingredients of WHS and placebo preparations, Table S2: Inclusion and exclusion criteria for recruitment of participants, Table S3: Baseline characteristics (vital signs and somatometry) of participants.
