# Supplementary material for: Oral Intake of Hydrangea serrata (Thunb.) Ser. Leaves Extract Improves Wrinkles, Hydration, Elasticity, Texture, and Roughness in Human Skin: A Randomized, Double-Blind, Placebo-Controlled Study
Source: Nutrients. 2020 May 28;12(6):1588. doi: 10.3390/nu12061588 (PMC7352416; doi:10.3390/nu12061588)
Supplement: Supplementary file 1 [file nutrients-12-01588-s001.pdf]

Supplementary Materials

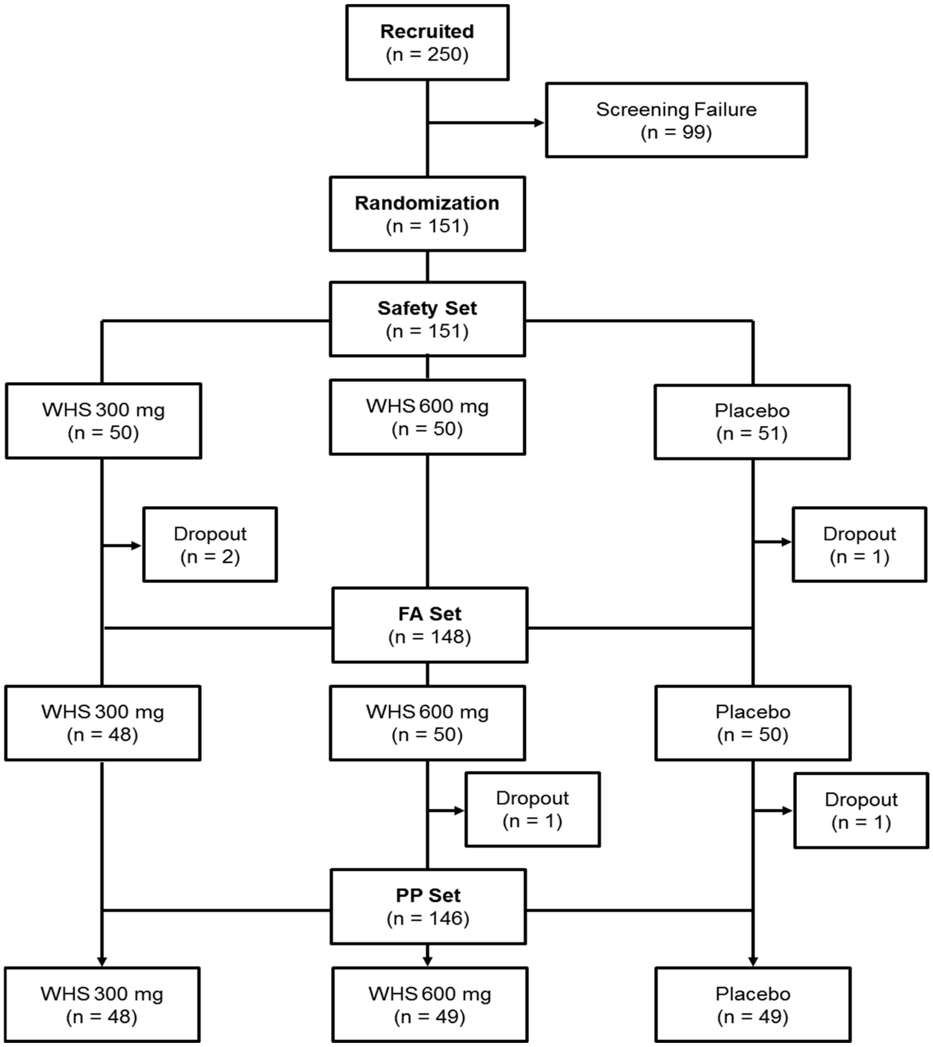

Figure S1. The study flow diagram

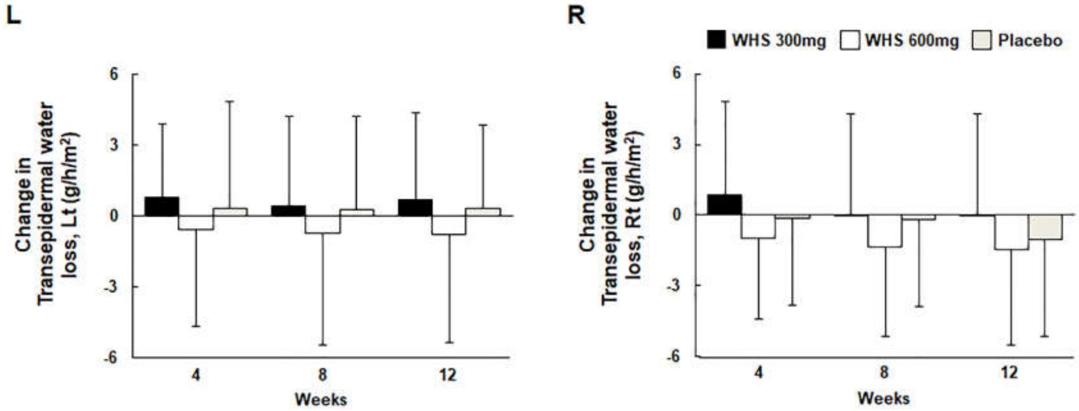

Figure S2. Changes in transepidermal water loss (TEWL) in taking WHS or placebo following 12 consecutive weeks. TEWL was measured with a Tewameter TM300.

Table S1. Ingredients of WHS and placebo preparations.

Ingredients	WHS 300 mg		WHS 600 mg		Placebo	
	Content (mg)	Content (%)	Content (mg)	Content (%)	Content (mg)	Content (%)
WHS	300.0006	33.3334	600.0003	66.6667	0	0
Silicon dioxide	15.3	1.7	15.3	1.7	13.6	1.7
Magnesium stearate	10.8	1.2	10.8	1.2	9.6	1.2
Microcrystalline cellulose	509.4945	56.61	209.4948	23.2772	245.9512	30.7439
Lactose	0	0	0	0	480	60
Hydroxypropyl methylcellulose	29.07	3.23	29.07	3.23	19.44	2.43
Carboxymethylcellulose calcium	27	3	27	3	24	3
Glycerol esters of fatty acids	0.693	0.077	0.693	0.077	0.616	0.077
Titanium dioxide	1.4697	0.1633	1.4697	0.1633	1.3064	0.1633
Monascus color	4.4082	0.4898	4.4082	0.4898	3.9184	0.4898
Caramel color	1.764	0.196	1.764	0.196	1.568	0.196
Total	900	100	900	100	800	100

Table S2. Inclusion and exclusion criteria for recruitment of participants.

**Inclusion criteria**
● Healthy male and female aged 35-60 years
● In visual assessment, over grade 3 of wrinkle in the crow's-feet area
● Those with an average score of 49 points or less when measured water retention on both cheeks using Corneometer
● Those who have agreed to participate before the start of the clinical trial and have signed a written informed consent
**Exclusion criteria**
● Those who suffer from or are currently undergoing treatment for skin diseases such as atopic dermatitis and psoriasis
● Those who are sensitive or allergic to pharmaceutical products or foods related to testing
● Those with abnormal skin conditions such as spots, acne, erythema, and capillary dilation in test areas
● Those who received treatment on the face within 1 month of the start of the test (skin peeling, chemical peeling, other skin care) or received treatment on the face within 6 months of the test, such as Botox and filler
● Those who use a steroid-containing skin external agent on the facial part or who have taken an oral retinoid/steroid agent within three months of the start of the test
● Those who used functional cosmetics for improving wrinkles (retinoids, retinol, AHA) or moisturizing cosmetics within 2 weeks of the start of the test
● Those who took diet pills (absorbent inhibitor and antidepressants, appetite suppressants, etc.), contraceptives or hormone medications, and diuretics within 1 month of the start of the test
● Those who have taken antioxidants, hyaluronic acid, health function foods containing collagen, evening primrose oil, medicine and health function foods containing vitamin A, C, and E within 2 weeks of the start of the test
● Those who AST (GOT) or ALT (GPT) is 120 IU/L or higher, or who γ-GTP is 180 IU/L or higher
● Those who creatinine is greater than 2.4 mg/dL
● Those who TSH is 0.1 μIU/mL or less and 10 μIU/ml or more
● Unregulated hypertension patients (160/100mmHg or more, based on measurement after 10 minutes of stabilization)
● Diabetes patients who blood sugar is not regulated (over 180 mg/dl of fasting blood sugar)
● Mental patients with schizophrenia, depression, drug addiction, and etc.
● Those who are pregnant, nursing, or planning to conceive within 3 months
● Those who are hospitalized, medicated, and undergoing rehabilitation due to alcohol use disorder, heart disease, and central nervous disorder through a history survey
● Smokers or those who have not smoked for less than a year
● Those who participated in other clinical trial within 1 month of the start of the test or have plans to participate during the test period
● Those who participated in the same clinical trial within 6 months of the start of the test or have plans to participate during the test period
● Those who are deemed inappropriate for the test

Table S3. Baseline characteristics (vital signs and somatometry) of participants.

Variable	WHS 300 mg group (*n* = 50)	WHS 600 mg group (*n* = 50)	Placebo group (*n* = 51)	*p*-Value[&]
	**Mean (SD)**	**Mean (SD)**	**Mean (SD)**	
**Age (years)**	48.31 (5.38)	49.43 (5.04)	48.31 (6.42)	0.5290
Weight (kg)	60.47 (8.68)	61.63 (11.44)	60.37 (9.24)	0.7767
Systolic bp[a)] (mmHg)	121.62 (15.06)	117.56 (14.26)	120.67 (11.72)	0.3056
Diastolic bp[a)] (mmHg)	73.62 (10.24)	71.10 (8.56)	73.59 (10.18)	0.3305
Pulse (times/min)	75.42 (8.58)	76.84 (10.16)	74.82 (9.27)	0.5412

a) Blood pressure. [&]: Compared between groups; *p*-value by One-Way ANOVA.
